# Silicosis among agate workers at Shakarpur: An analysis of clinic-based data

**DOI:** 10.4103/0970-2113.71955

**Published:** 2010

**Authors:** Nayanjeet Chaudhury, Ajay Phatak, Rajiv Paliwal, Chandra Raichaudhari

**Affiliations:** *Division of Chronic Diseases, Asian Institute of Public Health, Bhubaneswar, Orissa, India*; 1 *Department of Community Medicine, Central Research Services, Charutar Arogya Mandal, Karamsad, India*; 2 *Department of Pulmonary Medicine, PS Medical College, Karamsad, India*; 3 *Department of Radiology, PS Medical College, Karamsad, Gujarat, India*

**Keywords:** Silicosis, pneumoconiosis, tuberculosis, co-morbity

## Abstract

**Background:**

There is a high prevalence of silicosis and other morbid conditions leading to early death among agate workers at Khambhat of Gujarat.

**Aims:**

The present study describes the prevalence of X-ray positive silicosis in a sample of a high-risk group visiting a clinic at Shakarpur of Khambhat.

**Settings and Design:**

A cross-sectional study among 123 clinically suspected cases was conducted over 6 months.

**Materials and Methods:**

A chest physician and a radiologist independently evaluated the Chest X-rays of 123 clinically suspected patients of silicosis. Silicosis was confirmed if either of them rated the X-ray as positive.

**Statistical Analysis:**

Descriptive statistics and logistic regression were done using SPSS software version 14.

**Results:**

Out of 123 cases, 85 (69.1%) were confirmed as silicosis. There was no significant difference in the prevalence between males (70.3%) and females (69.4%). Workers with more than 10 years of exposure to silica had an odd ratio of 4.8, 95% CI (1.76, 13.60) compared to those with less than 10 years of exposure. A logistic regression analysis showed that for every extra year of exposure, the odds of getting silicosis increased by about 12%.

**Conclusions:**

This study highlights the catastrophic effects of exposures to silica in agate worker, which calls for urgent protective measures for this population.

## INTRODUCTION

Silicosis causes fibrosis and destruction in the lung that proves fatal in due course of time.[1] It is one of the world’s oldest known diseases that continues to kill people in occupations where workers are exposed to respirable fine dust over a long period. In India, an estimated three million workers are exposed to silica in mines and in industries, such as stone cutting, silica milling, agate, slate pencil, etc.[2] Among these, the highest prevalence of silicosis is in the slate pencil industry (54.5%) followed by workers in agate industries (38%).[3] High mortality rates due to silicosis is especially true of unorganized sector[4] probably because of comorbidities such as tuberculosis and malnutrition. However, studies on silicosis in the unorganized sector, especially in a setting like India, are scarce. In the unorganized sector, small stone polishing units that are based in the homes of workers do not fall under the purview of the factories act and are, therefore, not accessible for monitoring to regulatory authorities such as the factory inspectorate. In addition, since the work is done at home, primary workers and their family members are always at risk of silicosis due to the exposure to respirable dust.[5]

Studies conducted earlier reveal that the concentration of respirable dust in these household industries is very high and contain more than 60% free silica in it. Besides high prevalence of silicosis among the workers engaged in this unorganized industry, nonoccupationally exposed groups are also reported to have silicosis (6.8–11.8%), silicotuberculosis (2.8–6.3%), and tuberculosis(18.8–20.1%).[6]

This situation is true of the Shakarpur area of Khambhat, a coastal city of Gujarat, India, where several small agate stone polishing units operate from individual houses for many decades (Census of India, 1960).[7] However, there is no systematic documentation of patients’ records to date. Workers manufacture ornaments, decorative and showcase items from agate and other stones in their households. Even though silicosis is a notifiable disease as per factories act 1948,[8] the provisions of the Act are applicable only to the registered units in an organized sector. There is no system in place for medical professionals to document and inform government public health systems about the morbidity and mortality associated with silicosis.

To address the gap in knowledge with regard to the prevalence of silicosis in the unorganized agate industry at Shakarpur, a program was designed to identify symptomatic patients at a clinic in the area, the outcome of which is presented here as a study. The clinic was established by a medical college teaching hospital in collaboration with an NGO, which engages few ex-workers of agate polishing units as volunteers to help the victims with their rights for health and compensation. Chest physicians from the teaching hospital visited the clinic fortnightly to examine and treat symptomatic patients of silicosis.

## OBJECTIVE

The present study, conducted among the chest symptomatic patients of the clinic at Shakarpur, describes the prevalence of X-ray positive silicosis, in a sample of a high-risk group visiting the Shakarpur clinic.

## SUBJECTS AND METHODS

*Sample*. The study was carried out among 160 consecutive symptomatic patients who attended the health clinic at Shakarpur from June to December 2007.

### Setting

Three volunteers, who had worked in the agate industry, were trained to identify and recruit symptomatic patients based on inclusion and exclusion criteria (see below). In case of a nonambulatory patient, the physicians visited the patient at his/her residence for examination and treatment.

### Inclusion/exclusion criteria

The inclusion criteria for this study were patients above 15 years of age of both sexes living in Shakarpur, with symptoms of chronic cough for more than 3 months and/or breathlessness and a history of exposure to silica for more than a year. The eligible patients were examined by the chest physicians and referred for chest X-ray.

A caveat of note is that symptomatic patients who were unable to go to the clinic for clinical assessment and subsequently for chest X-ray due to severity or other disability of the morbid condition were not included in the analysis of the data presented below. However, they were visited by the physicians for symptomatic treatment.

## STUDY PROCEDURES AND MEASURES

A clinical assessment form was filled up by the physician following history taking and clinical examination. This was facilitated by the volunteers, especially in case of debilitated patients who could not give detailed history or who did not have an attendant. The purpose of the clinic and the documentation of the clinical profile of the patient were explained to the participant by the physician and the accompanying volunteer. No written consent was obtained from the participant as it was a routine clinical care program. However, in order to disseminate to scientific community and concerned individuals, the findings related to the morbidity among the workers, the research team obtained waiver of informed consent from the institutional ethics committee.

*Chest X-ray*. Of the 160 suspected patients identified by the PTRC volunteers based on inclusion criteria, 132 cases were clinically suspected to have silicosis by the chest physicians at the clinic and were referred for chest X-ray at a local hospital using a 300-mA machine, sponsored by the Lions’ Club of Khambhat. Nine of the 132 patients could not present in the hospital for the X-ray due to severe debilitation. The X-rays were examined independently by a senior radiologist and a senior chest physician. The X-rays were evaluated for the presence or absence of silicosis based on the presence or absence of fibrosis and small or large opacities in lung fields. The experts rated each X-ray as either positive or negative or undecided. Patients were diagnosed to have silicosis if either of the chest physician and/or the radiologist rated them as positive for small or large regular opacities in the chest X-ray. The overall Kappa for inter-rater agreement between the chest physician and the radiologist was 0.59. On grouping the undecided cases as negative, the Kappa increased to 0.786.

*Analysis*. Statistical Package of Social Sciences (SPSS) version 14^th^ was used to analyze the data. Odds ratio was used to show the effect of duration on the prevalence of silicosis. Logistic regression was employed to derive age-adjusted odds ratio. The reliability between the X-ray diagnosis by the physician and the radiologist was tested using Kappa statistics.

## RESULTS

### Demographic profile

Among the study participants [Table 1], there were 48 females and 74 males. There were more males with chest symptoms than females. Further, about 77% were in the age group 15–35 years whereas only 3.3% people were in the age group 60 years and above. In the age group 15–30 years, there were two cases that were below 18 years. The age and sex distribution of confirmed silicosis patients based on X-ray findings was similar to the above [Table 2].

**Table 1:** Age-sex distribution (frequency and percentage) of all participants (*N* = 122*)

Age groups (yrs)	Gender		Total
	Female	Male	
15–30	9 (18.8)	18 (24.3)	27 (22.1)
31–45	28 (58.3)	39 (52.7)	67 (54.9)
46–60	9 (18.8)	15 (20.3)	24 (19.7)
61–75	2 (4.2)	2 (2.7)	4 (3.3)
Total	48 (100)	74 (100)	122*(100)

* The age of one participant was missing

**Table 2:** Age-sex distribution (frequency, percentage) of confirmed cases (*N* = 85)

Age groups (yrs)	Gender		Total
	Female	Male	
15–30	6 (18.2)	9 (17.3)	15 (17.6)
31–45	19 (57.6)	32 (61.5)	51 (60.0)
46–60	7 (21.2)	11 (21.2)	18 (21.2)
61–75	1 (3.0)	0 (0)	1 (1.2)
Total	33 (100)	52 (100)	85 (100)

### Exposure history

Adequate exposure history could be elicited for 108 participants only. More than 40% of the patients (*N*=108) were found to have been working in the industry for more than 10 years. Four of them had been working for more than 30 years. Table 3 shows that with longer duration of exposure, progressively higher prevalence of X-ray-positive silicosis is seen among the patients.

**Table 3:** Duration of exposure and X-ray finding

Diagnosis	Duration of exposure		
	Up to 10 years	More than 10 years	Total
Positive	25 (55.6)	54 (85.7)	79 (73.1)
Negative	20 (44.4)	9 (14.3)	29 (26.9)

Odds ratio = 4.8, 95% CI (1.76, 13.60); Figures in parenthesis are in percentage

Although the odds ratio is very significant, it may be due to the obvious confounder, i.e., “age.” Logistic regression analysis with duration of exposure and age as “continuous” independent variables estimated the adjusted odds ratio to be 1.12 (95% CI (1.03, 1.21, *P*=0.005). Thus for every extra year of exposure, the odds of getting silicosis increased by about 12%.

### Chest X-ray

The chest physician could not decide the presence or absence of silicosis in 13 cases, while the radiologist was undecided about 22 cases. Although the overall agreement between the two experts was 0.59 [Table 4], when these doubtful cases were grouped as negative the Kappa increased considerably to attain the value of 0.786 [Table 5]. There was no significant difference in the X-ray positivity between males (70.3%) and females (69.4%).

**Table 4:** Agreement between X-ray reading by radiologist and chest physician for the presence or absence of silicosis

Diagnosis by the radiologist				
Diagnosis by the chest physician	Positive	Negative	Undecided	Total
Positive	74	0	7	81
Negative	3	17	9	29
Undecided	2	5	6	13
Total	79	22	22	123

Kappa = 0.59

**Table 5:** Agreement after grouping undecided cases as negative

Diagnosis by the radiologist			
Diagnosis by the chest physician	Positive	Negative	Total
Positive	74	7	81
Negative	5	37	42
Total	79	44	123

Kappa = 0.786

## DISCUSSION

Overall, 85 out of 123 cases (69.1%) were confirmed as silicosis by the physicians based on the X-ray reading and supported by clinical examination. After exclusion of 12 cases with uncertain exposure history, the prevalence among symptomatic workers with a clear history of exposure rose to 73.15%. This is not generalizable to the community at large of exposed workers as this was a clinic-based study conducted in survivor population, and there were some who were too ill to come to the clinic, hence not included in the analysis. However, it is of concern that three-fourths of all respiratory cases presenting in the clinic were diagnosed as silicosis. Additionally, comorbidities such as bronchial asthma, chronic bronchitis, tuberculosis, etc. that have been documented in agate workers in the past[6] also exist among the patients presented at the clinic. It is also likely that severe cases of respiratory morbidity were recruited because debilitated patients with a history of exposure to silica were more likely to access the services of the clinic.

The study corroborates the fact that longer duration of cumulative exposure to free silica is associated with higher prevalence of silicosis.[9] Six months after the clinic opened, 20 (12%) out of the 160 patients enrolled in the clinic died of silicosis.Of these 20 cases who died, 15 were in the age group 15–45 years and 5 patients between the ages of 46–60 years. This death distribution is similar to a cohort study conducted with 33,640 Chinese workers exposed to silica dust for at least 1 year, where only 25% of them were found to live beyond 33 years of age.[10] Thus, our finding, though based on a small clinic-based sample, supports early mortality of agate workers. Possibly, the extent of debilitation among our study participants is explained by the higher prevalence of silicosis among those who had more than a decade of exposure to the dust.

This study documents the severe respiratory morbidity and mortality of symptomatic silicosis victims, based on the consultants’ evaluations recorded in the clinic at Shakarpur. Also, it is noteworthy that a quarter of those with symptoms had no radiographic evidence of silicosis. It would be worthwhile to study the illnesses associated with silicosis mainly because some of them may be treatable such as obstructive airway disease, tuberculosis, and malnutrition. An adequate history of smoking may be helpful in explaining the obstructive airway diseases prevalent among the victims.

The agate stone polishing units at Shakarpur are part of the cottage industry sector, mostly a family traditional business. Thus other people in the family not working directly are also exposed to environmental exposure. The day care center for the children of the agate workers run by PTRC, Vadodara, is an example of primary prevention because it separates children of workers from possible dust exposure during working hours. These children may be observed for a medical surveillance by the clinic for further research.

The troubled community working in this industry knows the consequences but still continues to work with agate in order to earn its livelihood. Bonded labor among similar workers, especially among children in stone quarries, have been reported earlier.[11] The situation in Shakarpur is no different. Many people denied that they were currently working in agate industry, despite their occupation being known to the volunteers of the NGO. Our findings provide evidence of the catastrophic effects of exposures to silica in agate worker, which calls for urgent protective measures.
